# NKp30^+^ NK cells are associated with HBV control during pegylated-interferon-alpha-2b therapy of chronic hepatitis B

**DOI:** 10.1038/srep38778

**Published:** 2016-12-12

**Authors:** Xiaokun Shen, Binqing Fu, Yanyan Liu, Chuang Guo, Ying Ye, Rui Sun, Jiabin Li, Zhigang Tian, Haiming Wei

**Affiliations:** 1 Institute of Immunology and the CAS Key Laboratory of Innate Immunity and Chronic Disease, School of Life Sciences and Medical Center, University of Science and Technology of China, Hefei, Anhui 230027, China; 2 Hefei National Laboratory for Physical Sciences at Microscale, University of Science and Technology of China, Hefei, Anhui 230027, China; 3 Department of Infectious Diseases, First Affiliated Hospital of Anhui Medical University and Chaohu Hospital of Anhui Medical University, Hefei, Anhui, China; 4 Collaborative Innovation Center for Diagnosis and Treatment of Infectious Diseases, State Key Laboratory for Diagnosis and Treatment of Infectious Diseases, First Affiliated Hospital, College of Medicine, Zhejiang University, Hangzhou, Zhejiang 310003, China

## Abstract

A pressing need exists for improved therapeutic options for chronic hepatitis B (CHB). Pegylated-interferon-alpha (Peg-IFN-α) achieves sustained off-treatment responses in many cases because of its direct anti-viral effects and regulation of the immune response. However, non-responsiveness to Peg-IFN-α is frequent, and the mechanism is poorly understood. In this study, we found that the frequency and absolute number of NKp30^+^ natural killer (NK) cells increased markedly, accompanied by enhanced CD107a and IFN-γ production, during Peg-IFN-α-2b monotherapy or combination therapy with adefovir dipivoxil in patients with CHB, especially in responders. The responders and non-responders differed in the frequency of polyfunctional IFN-γ^+^ CD107^+^ NK cells. In addition, the increase in NKp30^+^ NK cells was negatively correlated with the HBV viral load and plasma HBeAg. Moreover, it was found that IL-15 may contribute to the up-regulation of NKp30 on the NK cells, and this up-regulation *was not* induced *in vitro* by Peg-IFN-α-2b alone. However, in the non-responders, these NKp30^+^ NK cells were dysfunctional because of increased NKG2A expression, which partly explains the inactivation of NKp30^+^ NK cells and the reduced capacity of these cells to produce antiviral cytokines. These findings may provide a new mechanism to explain the variable efficacy of Peg-IFN-α-2b therapy.

Chronic hepatitis B (CHB) constitutes a major health care problem worldwide, with nearly 240 million people affected^1^. The available treatment options for CHB include interferon, pegylated interferon (Peg-IFN), and five oral nucleotide analogues (NAs)^2^. The anti-HBe seroconversion rates of current therapies are approximately 30% with Peg-IFN and 20% with NAs^3^,^4^. Only a minority of Peg-IFN-treated patients achieve sustained HBV control, suggesting that the clinical efficacy of Peg-IFN can be improved^5^,^6^. This improvement could be based on an enhanced understanding of the molecular mechanism by which HBV subverts antiviral immune responses. Furthermore, the immune mechanisms underlying the differences between responders and non-responders merit further investigation.

Innate immune effector cells, such as natural killer (NK) cells, provide the first line of defence against viral infection^7^. Several findings prompted us to study NK cells in the context of hepatitis B treatments. First, 30–50% of intrahepatic lymphocytes are NK cells^8^. Second, the current treatment regimen for CHB includes Peg-IFN-α, a potent activator of NK cells^9^. Third, the genotype for certain receptors regulating NK cell activity has been associated with treatment-induced HBV clearance^10^,^11^.

In humans, NK cells differentiate into two subsets: (1) CD56^dim^ NK cells, which mediate cytotoxicity and represent the majority (80–90%) of NK cells in normal individuals; and (2) CD56^bright^ NK cells, which secrete large amounts of cytokines, such as IFN-γ and TNF-α, and account for 5–10% of all NK cells^12^. Individual NK cells display a diverse repertoire of activating and inhibitory receptors, including killer immunoglobulin-like receptors, natural cytotoxicity receptors (NKp30, NKp44, and NKp46), and c-lectin receptors (NKG2A/CD94, NKG2C/CD94, NKG2D, and CD161)^13^. The summation of activating signals, when not opposed by inhibitory signals, leads to the release of granzymes and perforin and results in target cytolysis^14^. The current study aims to investigate the immunoregulatory role of Peg-IFN-α-2b regarding NK cells and viral clearance in HBeAg-positive CHB patients undergoing Peg-IFN-α-2b treatment.

The present study evaluated the relationship between NK cells and the response to Peg-IFN-α-2b treatment. A maintenance of the number and recovery of the function of circulating NKp30^+^ NKG2A^−^ NK cells were found to be closely correlated with a better response to Peg-IFN-α-2b treatment. In contrast, CHB patients who failed to respond to Peg-IFN-α-2b treatment had increased numbers of NKp30^+^ NKG2A^+^ NK cells, indicating that NKG2A was responsible for the dysfunction of the NKp30^+^ NK cells in the non-responders. Overall, the current study explores the important role of NKp30^+^ NKG2A^−^ NK cells in CHB therapy and extends our understanding of the immunoregulatory role of Peg-IFN-α-2b regarding NK cells.

## Results

### Alteration in the numbers of NK cells, especially the CD56^bright^ NK subset, is associated with treatment response

**Figure 1:**
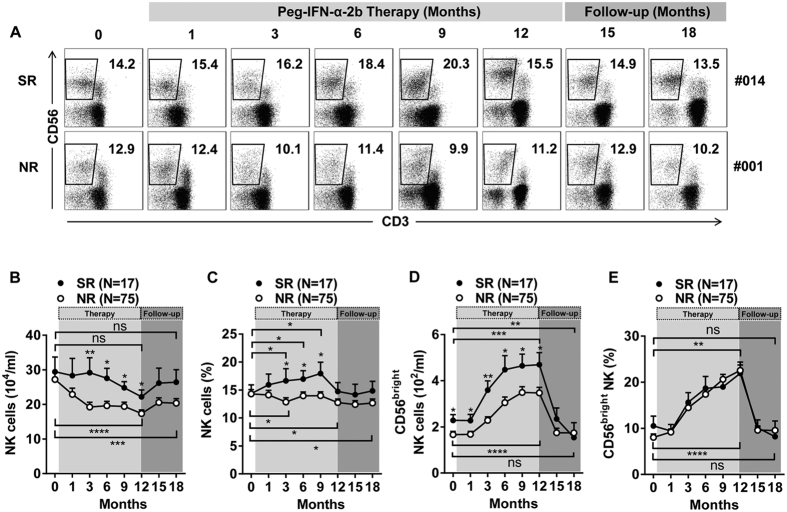
Peg-IFN-α-2b increased CD56^bright^ NK cell numbers throughout the course of treatment. **(A)** Longitudinal analysis of CD3^−^ CD56^+^ NK cell frequency in the SR and NR patients. **(B)** Absolute numbers of NK cells during Peg-IFN-α-2b therapy and follow-up in the SR and NR patients. **(C)** NK cell frequencies during Peg-IFN-α-2b therapy and follow-up in the SR and NR patients. **(D)** Absolute numbers and **(E)** frequencies of CD56^bright^ NK cells during Peg-IFN-α-2b therapy and follow-up in the SR and NR patients. Horizontal bars indicate the mean values with the standard error of the mean. Analyses of unpaired data were performed using the Mann-Whitney *U*-test, and the paired data were analysed with the Wilcoxon matched pairs test. Significant changes are marked with asterisks, *p < 0.05, **p < 0.01, ***p < 0.001, ****p < 0.0001.

To elucidate the role of NK cells in Peg-IFN-α-2b-treated HBeAg-positive patients, the NK cells were first defined as single cells within the lymphocyte gate, i.e., the cells were CD56^+^ yet 7-AAD^−^, CD3^−^, CD14^−^, and CD19^−^ to exclude dead cells, T cells, monocytes, and B cells, respectively (Supplementary Figure 2A). We longitudinally analysed and compared the frequencies (Fig. 1A) and absolute numbers (Fig. 1B) of NK cells in SR patients and NR patients. The absolute numbers of NK cells in the SR patients were higher than in the NR patients from 3 to 12 months (Fig. 1B). Compared to baseline, there was a statistically significant progressive decline in the absolute numbers of NK cells in the NR patients, but this decline was not observed in the SR patients (Fig. 1B). During treatment with Peg-IFN-α-2b, the SR patients gained a higher proportion of NK cells than did the NR patients beginning at 3 to 9 months (Fig. 1C). Compared to baseline, there was a statistically significant increase in the proportion of NK cells in the SR patients starting at 3 months (Fig. 1C). A different trend was found in the NR subjects, in which a statistically significant decline in the proportion of NK cells occurred that began at 3 months and continued throughout follow-up (Fig. 1C). Moreover, treatment with adefovir dipivoxil (ADV) resulted in similar trends in the percentages and absolute numbers of NK cells (Supplementary Figure 2B,C). Additionally, we detected two subsets of NK cells during Peg-IFN-α-2b therapy. The absolute numbers and percentages of CD56^bright^ NK cells significantly increased during Peg-IFN-α-2b therapy (Fig. 1D,E). Moreover, the absolute numbers of CD56^bright^ NK cells in the SR patients were higher than those in the NR patients from 0 to 12 months (Fig. 1D).

### Persistently increased NKp30^+^ NK cell numbers were found in patients throughout the course of Peg-IFN-α-2b therapy, especially in SR patients

**Figure 2:**
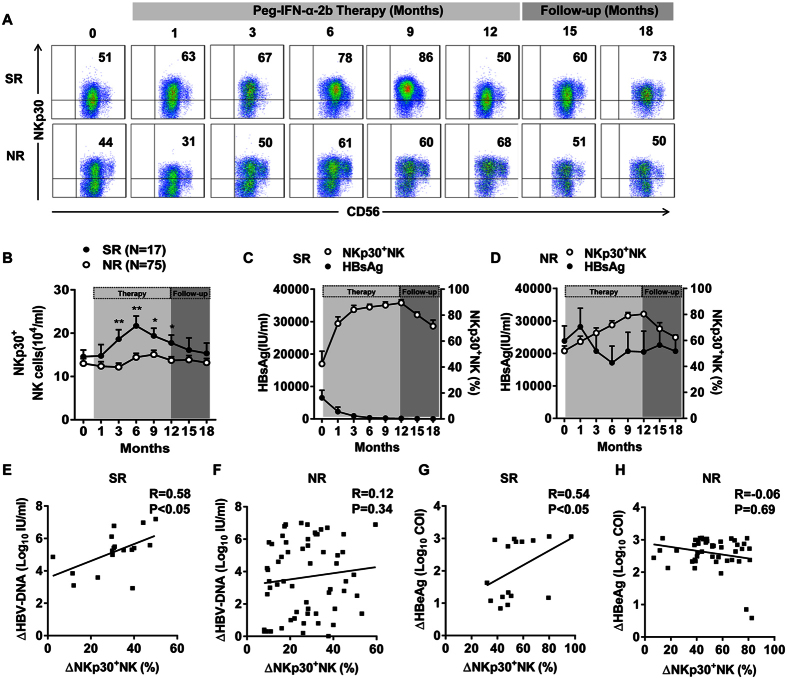
Peg-IFN-α-2b treatment persistently increased NKp30 expression on NK cells, especially in the SR patients, which correlated with clinical outcomes. (**A**) Representative dot plots show NKp30 expression on NK cells upon treatment in a representative patient. (**B**) Absolute numbers of NKp30^+^ NK cells during Peg-IFN-α-2b therapy and follow-up in the SR and NR patients. (**C,D**) Cumulative longitudinal data of the plasma HBsAg concentration (IU/mL) and the percentage of NKp30^+^ NK cells in the SR and NR patients. (**E,F**) Spearman’s rank correlation coefficient between the change in log_10_ HBV-DNA and the change in NKp30^+^ NK cells was determined. (**G,H**) Spearman’s rank correlation coefficient between the change in log_10_ HBeAg and the change in NKp30^+^ NK cells was determined. ΔLog_10_ HBV-DNA (IU/ml) = Log_10_ HBV-DNA_baseline_ (IU/ml) − Log_10_ HBV-DNA_12 months_ (IU/ml); ΔNKp30^+^ NK cell (%) = NKp30^+^ NK cell (%)_12 months_ − NKp30^+^ NK cell (%)_baseline_; ΔLog_10_ HBeAg (COI) = Log_10_ HBeAg (COI)_baseline_ − Log_10_ HBeAg (COI)_12 months_. Horizontal bars indicate the mean values with the standard error of the mean. Analyses of unpaired data were performed using the Mann-Whitney *U*-test, *p < 0.05, **p < 0.01.

Next, we investigated the influence of Peg-IFN-α-2b on the dynamics of the NK cell phenotypes. We analysed the proportion of natural cytotoxicity receptor (NCR)-expressing NK cells at different time points. The percentage of NK cells expressing the activating NCR NKp30 significantly increased (Fig. 2A). Notably, the absolute number of NKp30^+^ NK cells was significantly higher in the SR patients than in the NR patients from 3 to 12 months (Fig. 2B). However, the expression of other NCRs (NKp44 and NKp46) was not significantly different between the SR and NR patients during treatment (Supplementary Figure 3A,B). Furthermore, combination therapy with ADV also did not affect the expression of other NCRs on NK cells (Supplementary Figure 4A–C). To further confirm the relationship between NKp30^+^ NK cells and virological outcome, we analysed the clinical data from the longitudinal cohort. We found a more substantial reduction in HBsAg in the SR patients than in the NR patients (Fig. 2C,D). A Spearman’s rank correlation coefficient between the decrease in log_10_ HBV-DNA (or HBeAg) and the increase in NKp30^+^ NK cells was determined at 12 months. We found that the decrease in HBV-DNA and HBeAg was correlated with the therapy-induced increase in NKp30^+^ NK cells in the SR patients but not in the NR patients (Fig. 2E–H).

### Peg-IFN-α-2b potently increased NKp30^+^ NK cell proliferation by promoting IL-15 production

**Figure 3:**
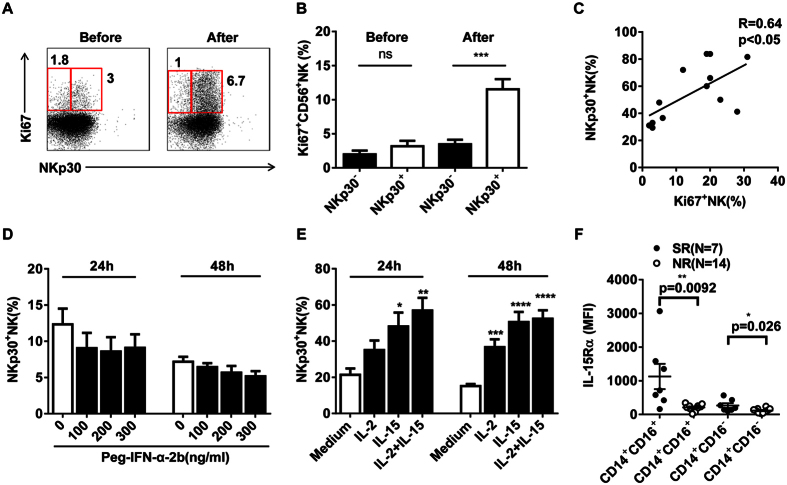
IL-15 drives NKp30^+^ NK cell proliferation, which could not be induced by Peg-IFN-α-2b. (**A**) The proportion of NKp30^+^ NK cells expressing Ki-67 before and after the start of Peg-IFN-α-2b therapy in a representative patient. (**B**) Ki-67 expression was significantly higher on NKp30^+^ NK cells after the start of Peg-IFN-α-2b therapy than at baseline. (**C**) The relationship between the percentage of Ki67^+^ NK cells and NKp30^+^ NK cells. The Spearman correlation coefficient (r) and p-values are indicated. (**D**) The percentage of NKp30^+^ NK cells was not altered by Peg-IFN-α-2b treatment. (**E**) The percentage of NKp30^+^ NK cells was altered by stimulation with IL-2 (100 U/ml), IL-15 (10 ng/ml), or IL-2 (100 U/ml) plus IL-15 (10 ng/ml). **(F)** The mean fluorescence intensity (MFI) of IL-15Rα (CD215) expression on two subsets of monocytic cells from the SR (black) and NR (white) patients. Horizontal bars indicate the mean values with the standard error of the mean. Analyses of unpaired data were performed using the Mann-Whitney *U*-test, and analyses of paired data were performed using the Wilcoxon test, *p < 0.05, **p < 0.01, ***p < 0.001, ****p < 0.0001.

To investigate whether the increase in circulating NKp30^+^ NK cells was due to enhanced proliferation, Ki-67 expression was examined. Ki-67 expression was significantly higher in NKp30^+^ NK cells after the start of the Peg-IFN-α-2b therapy than at baseline (Fig. 3A,B). Moreover, NKp30 expression on NK cells was related to the expression of Ki-67 (Fig. 3C). In murine models, NK cell proliferation is predominantly driven by IL-15, which is required by IFN-α^15^. We next investigated whether the observed increase in NKp30 expression on NK cells was induced by Peg-IFN-α-2b or IL-2/IL-15 *in vitro*. In the current study, NKp30 expression on NK cells was greatly enhanced by IL-15 but could not be induced *in vitro* by Peg-IFN-α-2b stimulation for either 24 or 48 h (Fig. 3D,E). Next, we investigated which cells were responsible for the production of IL-15. Biologically active IL-15 is presented on the cell surface as a complex with the IL-15Rα chain (CD215)^16^,^17^. We observed higher levels of CD215 (IL-15Rα) on the pro-inflammatory CD14^+^ CD16^+^ monocytes and classical CD14^+^ CD16^−^ monocytes than on the other lymphocyte subsets examined (Supplementary Figure 5). Furthermore, in the SR patients, the CD215 expression on these two subsets of monocytic cells was significantly higher than that in the NR patients (Fig. 3F). These data suggest that monocyte-derived IL-15 plays an important role in regulating the capacity of NK cells to proliferate more rapidly in SR patients than in NR patients.

### Peg-IFN-α-2b therapy maintained the anti-viral function of NKp30^+^ NK cells in SR patients

**Figure 4:**
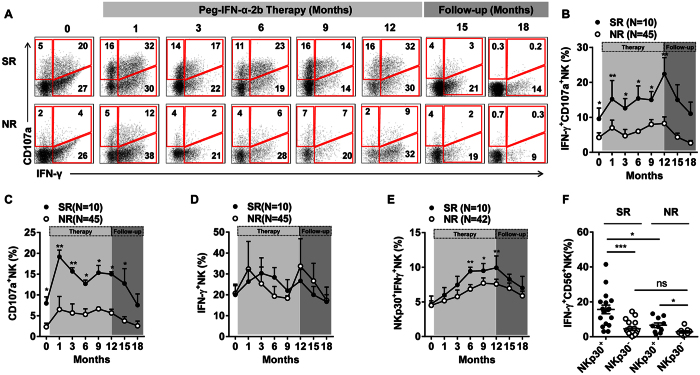
Peg-IFN-α-2b regulated NK cell function by increasing degranulation and IFN-γ expression, which correlated with clinical outcomes. (**A**) Longitudinal analysis of IFN-γ and CD107a co-expression by NK cells in the SR and NR patients. (**B–D**) The frequencies of IFN-γ^+^ CD107a^+^ NK, CD107a^+^ NK, and IFN-γ^+^ NK cells during Peg-IFN-α therapy and follow-up. (**E**) Longitudinal analysis of IFN-γ expression by NKp30^+^ NK cells in the SR and NR patients. (**F**) IFN-γ expression by NKp30^+^ NK cells in the SR and NR patients at the end of Peg-IFN-α therapy. Horizontal bars indicate the mean values with the standard error of the mean. Analyses of unpaired data were performed using the Mann-Whitney *U*-test, and analyses of paired data were performed using the Wilcoxon test, *p < 0.05, **p < 0.01, ***p < 0.001.

Previous studies in patients with CHB have shown that the increased population of CD56^bright^ NK cells maintained their cytotoxic potential but were impaired in their capacity to produce the antiviral cytokine IFN-γ^18^. Therefore, we determined whether the increased subset of NKp30^+^ NK cells induced by Peg-IFN-α-2b therapy displayed this type of impairment in effector function. To investigate the dynamics of alterations in NK cell effector functions, we analysed the proportion of NK cells expressing IFN-γ and CD107a using PMA/Ion as a stimulant *in vitro* (Fig. 4A). There was a significant difference in the frequency of polyfunctional IFN-γ^+^ CD107^+^ NK cells in the responders and non-responders (Fig. 4B). Furthermore, the proportion of NK cells expressing CD107a was also higher in the SR patients (Fig. 4C). However, the IFN-γ-expressing NK cells were not different between the SR and NR patients (Fig. 4D). Together, these results indicate that NK cells co-expressing IFN-γ and CD107a and CD107a-expressing NK cells were beneficial to CHB patient in the response to Peg-IFN-α-2b therapy. To determine the function of the NKp30^+^ NK cells in the SR and NR patients, PBMCs were stimulated with IL-12 *ex vivo*. We found that the percentage of NKp30^+^ IFN-γ^+^ NK cells was significantly higher in the SR patients than in the NR patients from 6 to 9 months (Fig. 4E). Furthermore, we examined differences in the IFN-γ-producing NKp30^+^ NK cells between the SR and NR patients at the end of therapy. As expected, the percentage of IFN-γ-producing NKp30^+^ NK cells was higher in the SR patients than in the NR patients (Fig. 4F).

### SR patients gained CD62L^+^ NKp30^+^ NK cells during Peg-IFN-α-2b therapy

**Figure 5:**
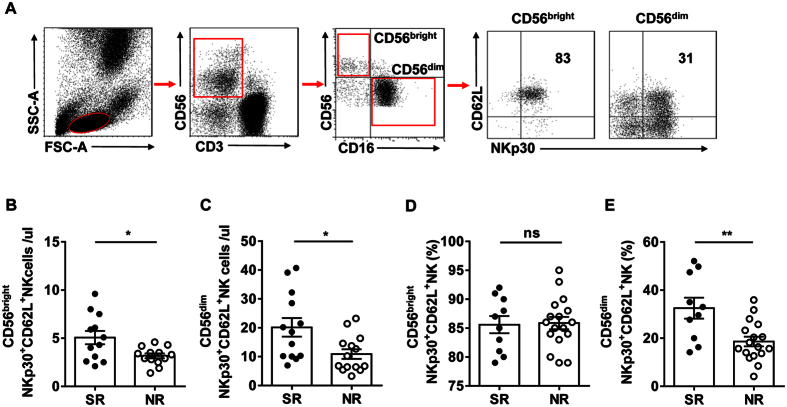
SR patients had more CD62L^+^ NKp30^+^ NK cells than NR patients at the end of Peg-IFN-α-2b therapy. (**A**) Gating strategy. (**B,C**) Absolute numbers of CD56^btight^ CD62L^+^ NKp30^+^ NK and CD56^dim^ CD62L^+^ NKp30^+^ NK cells in the SR patients and NR patients after the start of Peg-IFN-α therapy. (**D,E**) The percentage of CD56^dim^ CD62L^+^ NKp30^+^ NK cells was higher in the SR patients than in the NR patients after the start of Peg-IFN-α-2b therapy. Horizontal bars indicate the mean values with the standard error of the mean. Statistical analyses were performed using the Mann-Whitney *U*-test, *p < 0.05; **p < 0.01.

We next investigated whether the increased IFN-γ-producing NKp30^+^ NK cell population was related to CD62L. We analysed the proportion and total numbers of NKp30^+^ NK cells expressing CD62L in CD56^bright^ and CD56^dim^ populations (Fig. 5A). At the end of therapy, the total numbers of CD56^bright^ NKp30^+^ CD62L^+^ and CD56^dim^ NKp30^+^ CD62L^+^ NK cells were higher in the SR patients than in the NR patients (Fig. 5B,C). Furthermore, the percentage of CD56^dim^ NKp30^+^ CD62L^+^ NK cells was significantly higher in the SR patients at the end of treatment (Fig. 5D,E). These results indicate that more CD56^bright^ NKp30^+^ NK cells and CD56^dim^ NKp30^+^ NK cells exhibited the CD62L phenotype after the start of Peg-IFN-α therapy, which is beneficial for producing IFN-γ.

### Peg-IFN-α-2b increased NKG2A expression on NKp30^+^ NK cells, which negatively affected the clinical outcome

**Figure 6:**
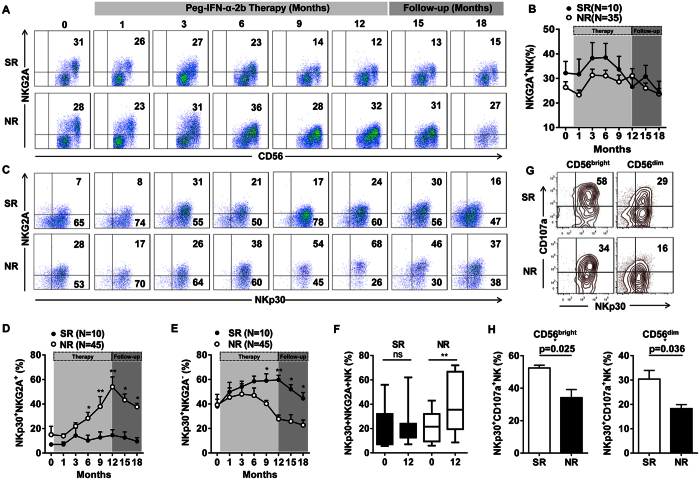
NKp30^+^ NK cells exhibited a progressive increase in the expression of the inhibitory receptor NKG2A in the NR patients. (**A**) Changes in the percentage of NKG2A-expressing NK cells upon treatment in a representative patient. (**B**) A longitudinal analysis of the expression of NKG2A in paired samples of the SR and NR patients. (**C**) Changes in NKp30 and NKG2A co-expression on NK cells in a representative patient. (**D,E**) A longitudinal analysis of NKp30 and NKG2A co-expression on NK cells in paired samples of the SR and NR patients. (**F**) The percentages of NKp30^+^ NKG2A^+^ NK cells were significantly different between the SR and NR patients. **(G)** CD107a expression on CD56^bright^ NKp30^+^ and CD56^dim^ NKp30^+^ NK cells in the SR and NR patients after starting the Peg-IFN-α-2b therapy. **(H)** The percentage of CD56^bright^ NKp30^+^ CD107a^+^ and CD56^dim^ NKp30^+^ CD107a^+^ NK cells was higher in the SR patients than in the NR patients after starting the Peg-IFN-α-2b therapy. Horizontal bars indicate the mean values with the standard error of the mean. Statistical analyses were performed using the Mann-Whitney *U*-test, *p < 0.05, **p < 0.01.

Recently, we reported that HBV infection increased NKG2A expression on NK cells in mice and humans, reducing their ability to clear HBV^19^. Therefore, we investigated whether this impaired capacity to inhibit HBV was affected by an altered expression of inhibitory NK cell receptors in NR patients. The dynamics of NKG2A, Tim-3, and PD-1 expression on NK cells was observed. Tim-3 and PD-1 are two important receptors on exhausted HBV-specific CD8^+^ T cells^20^. In our study, the inhibitory receptor NKG2A was markedly down-regulated on NK cells after starting the Peg-IFN-α-2b therapy in the SR patients but not in the NR patients (Fig. 6A,B). There was no decrease in Tim-3 or PD-1 expression on NK cells during the Peg-IFN-α therapy (Supplementary Figure 3C,D). To determine whether Peg-IFN-α-2b could induce high levels of NKG2A expression on NKp30^+^ NK cells in NR patients, we compared the NKG2A expression on the NKp30^+^ NK cells between the SR and NR patients during therapy (Fig. 6C). We found that the percentage of NKG2A^+^ NKp30^+^ NK cells was higher in the NR patients than in the SR patients during Peg-IFN-α-2b therapy, especially at later periods (6, 9, and 12 months) and during follow-up (15 and 18 months) (Fig. 6D). In contrast, the percentage of NKG2A^−^ NKp30^+^ NK cells was higher in the SR patients than in the NR patients (Fig. 6E). Furthermore, this increase in the percentage of NKG2A^+^ NKp30^+^ NK cells was significant in the NR patients (Fig. 6F). The summation of inhibitory signals, when not opposed by activating signals, inhibited NK cell function. We hypothesized that a low level of NKG2A expression in combination with a high NKp30 level mediated a protective effect in the SR patients. To address this question, an *in vitro* co-culture experiment was performed. Purified NK cells from the SR or NR patients were co-cultured with K562 cells. We found a higher percentage of CD107a-expressing CD56^bright^ NKp30^+^ NK cells and CD56^dim^ NKp30^+^ NK cells in the SR patients when compared with the NR patients (Fig. 6G,H). In conclusion, the high level of NKG2A expression on NKp30^+^ NK cells may have inhibited the degranulation capacity of the NK cells in the NR patients, which negatively affected the clinical outcome.

## Discussion

In this study, we found an increase in CD56^bright^ NK cells during Peg-IFN-α-2b treatment (with or without ADV), which is in agreement with the Peg-IFN-α-2a-induced expansion of activated CD56^bright^ NK cells^21^. This is similar to the observed effect of IFNα therapy in HCV or HDV infection^22^,^23^. The combination of Peg-IFN-α-2b with ADV did not result in a higher rate of sustained virological or serological off-treatment response (Supplementary Table 1). Additionally, a persistent increase in NKp30^+^ NK cells was observed during the Peg-IFN-α-2b treatment course, which may be associated with clinical outcomes of the patients. This is consistent with the observed effect on NK cells of Peg-IFN-α and ribavirin therapy in HCV infection^24^. In addition, a recent study found a significant increase in NKp30 expression on CD56^bright^ NK cells with 6 months of Peg-IFN-α therapy onwards, and high levels of NKp30^+^ CD56^bright^ and CD56^dim^ cells were associated with lower HBsAg levels during sequential treatments with nucleos(t)ide analogues (NUCs)^25^. Moreover, the current study characterized the dynamics of circulating NKp30^+^ NK subsets and revealed the correlation of these subsets with reduced plasma viral DNA and HBeAg in HBeAg-positive CHB patients undergoing Peg-IFN-α-2b treatment.

Previous studies have demonstrated that CD56^bright^ NK cell proliferation was increased after utilizing combination therapy with Peg-IFN-α and an NA^10^. The ability of Peg-IFN-α therapy to induce IL-15 has been demonstrated^9^. Moreover, Richer *et al*. found that type I IFN is a major regulator of IL-15 expression in a PV (pichinde virus) infection mouse model^26^. In addition, in the current study, IL-15 was the possible cause of the increased NKp30^+^ NK cell frequency. When analysing Ki-67 expression in NK cells, we found an increase in Ki-67 expression in the NKp30^+^ NK cells. Furthermore, the IL-15Rα-expressing pro-inflammatory CD14^+^ CD16^+^ monocytes and classical CD14^+^ CD16^−^ monocytes differed between the SR and NR patients.

The responders to Peg-IFN-α-2b therapy exhibited an initially rapid increase in circulating IFN-γ^+^ CD107a^+^ NK cells compared with the NR patients during the Peg-IFN-α-2b treatment. During this process, the NKp30 expression on the NK cells significantly increased. Our data indicate that the NKp30^+^ NK cells exhibited an enhanced functional profile, as shown by the pattern of IFN-γ and CD107a co-expression.

Peg-IFN-α-2a monotherapy induced a shift from primary CD56^dim^ NK cells to more immature CD56^bright^ NK cells^9^. CD56^bright^ NK cells are known as the precursors of CD56^dim^ NK cells, which gradually differentiate from CD56^dim^ CD62L^+^ NK cells to terminally differentiated CD56^dim^ CD62L^−^ NK cells^27^. CD56^dim^ CD62L^+^ NK cells produce cytokines upon the engagement of activating receptors^27^. Moreover, our previous study indicated that, in murine models, CD62L was a marker for a mature NK cell subset in the liver and affected the magnitude of the local NK cell response to viral infection^28^. In the group under examination, we observed higher absolute numbers of CD56^dim^ NKp30^+^ CD62L^+^ NK cells in the responders than in the NRs at the end of treatment. This finding suggests that NKp30^+^ CD62L^+^ NK cells might play an important role in the control of HBV during Peg-IFN-α-2b therapy.

NKG2A^low^ NK cells have also been implicated in HCV protection and control^29^. Furthermore, higher levels of NKG2A expression on NK cells were demonstrable in HCV-infected patients who failed to achieve a sustained virologic response during Peg-IFN and ribavirin therapy^30^,^31^. In accordance with the previous study, the current data suggest that the selective NK cell functional defects observed following Peg-IFN-α-2b therapy might be attributable to the expression of the inhibitory receptor NKG2A on activated NKp30^+^ NK cells. We have previously demonstrated that the presence of the CD3^bright^ CD56^+^ T-cell population in CHB patients can be used as a novel immunological predictor before clinical Peg-IFN-α treatment^32^. In this study, we provide another indicator for evaluating the efficacy of Peg-IFN-α treatment of CHB patients during therapy in tandem.

In conclusion, the present data shed new light on the contrasting immunomodulatory effects of Peg-IFN-α-2b in NK cells *in vivo*. Significant differences were observed in the NK cell phenotypes and functions between the SR and NR patients. The imbalance in NK cell receptor expression and the dysfunction of NK cells during Peg-IFN-α-2b therapy may contribute to the lack of a Peg-IFN-α-2b response.

## Methods

### Patients

A total of 102 Chinese patients with HBeAg-positive CHB infection (without any antiviral treatment within the previous 6 months) were recruited for this study from June 2012 to July 2014. All patients were negative for anti-hepatitis C and anti-human immunodeficiency virus antibodies. The patients were randomly assigned to two different treatment groups and received standard of care treatment with Peg-IFN-α-2b (PegIntron, Schering-Plough Company) weekly (Group 1) or Peg–IFNα-2b weekly in combination with 10 mg adefovir dipivoxil (ADV, Hepsera, Gilead Sciences, Foster City, CA, USA) daily (Group 2) (Supplementary Figure 1). Peg-IFN-α-2b was administered subcutaneously (1.5 μg/kg body weight for up to 12 months). Before and after the start of Peg-IFN-α-2b treatment peripheral blood samples from the patients were obtained. From this group, 92 patients were selected (Supplementary Figure 1). Sustained response (SR) patients consisted of 17 responders, defined according to the European Association for the Study of the Liver guidelines^33^ as persistently undetectable HBeAg and a result of HBV DNA <2,000 IU/mL, with the development of antibodies to HBeAg (anti-HBe). The non-responder (NR) patients consisted of 75 individuals with no HBeAg clearance and without HBV DNA <2,000 IU/mL at the end of follow-up (Table 1).

### Ethics Statement

All patients gave written informed consent, and our study was approved by the ethics committee of the First Affiliated Hospital of Anhui Medical University (Grant No. K2010003). All the experiments were carried out in accordance with the approved guidelines and regulations, including any details. Clinical trial registration numbers were ChiCTR-TRC-12002226 and the URLs were http://www.chictr.org.cn/en/. Registered date was April 17, 2012.

### Surface staining of NK cells

For direct immunofluorescence staining of human whole blood, first, the appropriate volume of a fluorochrome-conjugated monoclonal antibody was added to 100 μL of fresh whole blood, which was then incubated in the dark at 4 °C for 30 min. A complete list of antibodies used for staining is available in Supplementary Table 2. Second, 2 mL of 1 × RBC Lysis Buffer (Biolegend) was added, and the solutions were incubated for 10 min in the dark at room temperature. Then, the samples were centrifuged at 350 × *g* for 5 min, and the supernatants were removed. After washing once, the solutions were analyzed using a FACSCalibur flow cytometer (Becton Dickinson), and the data were analyzed using FlowJo analysis software 7.6.1 (TreeStar, Inc.).

### Intracellular staining and the effector function of NK cells

Fresh peripheral blood mononuclear cells (PBMCs) were isolated by Ficoll-Hypaque (Solarbio, Beijing) density-gradient centrifugation. PBMCs (1 × 10^6^/mL) were cultured with RPMI 1640 supplemented with 10% fetal bovine serum in the presence of phorbol myristate acetate (PMA) (50 ng/mL) and ionomycin (1 μg/mL). One hour later, monensin (2.5 μg/mL) (all of the stimulators were purchased from Sigma–Aldrich, Gillingham, UK) and PE-anti-CD107a were added. The cells were harvested after culturing for 4 h at 37 °C and 5% CO_2_, and stained with APC-CY7-anti-CD3 and Alexa-647-anti-CD56 for 30 min at 4 °C in the dark. After undergoing the fixation and permeabilization processes, the cells were stained with FITC-anti–IFN-γ for 1 h at 4 °C in the dark, washed twice with a permeabilization buffer, and analyzed by flow cytometry. The appropriate isotypes of antibodies were used as controls. To measure the NKp30^+^ NK cells’ capacity to produce IFN-γ, PBMCs were incubated with rhIL12 (10 ng/mL, USTC) for 16 h at 37 °C, 5% CO_2_, with monensin (2.5 μg/mL) added for the last 4 h, and then stained with APC-CY7-anti-CD3, Alexa-647-anti-CD56, or PE-anti-NKp30 for 30 min at 4 °C in the dark; then, the PBMCs were permeabilized and stained for FITC-anti-IFN-γ. To assess proliferation, the NK cells were permeabilized and stained with Alexa-647-anti-Ki-67 directly *ex vivo*.

### NK cell purification

CD3^−^ CD56^+^ NK cells were enriched from PBMCs with an NK Cell Isolation Kit (Miltenyi Biotec). The purity of the NK cells, measured by flow cytometry, was greater than 95%.

### *In vitro* cell culture and stimulation experiment

A total of 1 × 10^5^ human NK cells were cultured at 37 °C in a 5% CO_2_ incubator. The cells were incubated in a medium, either alone or with Peg-IFN-α-2b (10 ng/mL; PegIntron, Schering-Plough Company), IL-2 (100 U/mL; Changchun Institute of Biological Products), IL-15 (10 ng/mL; PeproTech), or IL-15 (10 ng/mL) plus IL-2 (100 U/mL) for 24 or 48 h. For NK cells cytotoxicity detection *in vitro*, 1 × 10^6^ human NK cells and 1 × 10^5^ cells of the erythroleukemia cell line K562 (American Type Culture Collection) E/T = 10:1 were co-cultured for 4 h, and degranulation was detected with PE-anti-CD107a.

### Virological assessment

Plasma HBV DNA levels were quantified using a Roche COBAS TaqMan HBV test (Version: COBAS TaqMan 48 analyzer, F. Hoffmann-La Roche Ltd., Basel, Switzerland). Plasma HBsAg levels were quantified using a Roche Elecsys HBsAg II quant assay (Roche Diagnostics, China). The quantification of Plasma HBeAg and anti-HBe levels was performed based on the cut-off index (COI) by a Roche Elecsys 2010 immunoanalyzer (Roche Diagnostics, China). ALT was measured locally in accordance with standard procedures. HBV genotype was carried out using genotype-specific primers^34^.

### Statistical analyses

All data were presented as the mean ± SEM, and were analyzed using GraphPad 6 software. For paired data, the Wilcoxon signed rank test was used; for non-paired data, the Mann-Whitney *U* test was utilized. For analysis of correlation, the Spearman correlation coefficient was calculated. Significant differences were defined as p < 0.05.

## Additional Information

**How to cite this article**: Shen, X. *et al*. NKp30^+^ NK cells are associated with HBV control during pegylated-interferon-alpha-2b therapy of chronic hepatitis B. *Sci. Rep.* **6**, 38778; doi: 10.1038/srep38778 (2016).

**Publisher's note:** Springer Nature remains neutral with regard to jurisdictional claims in published maps and institutional affiliations.

## Supplementary Material

Supplementary Information

## Figures and Tables

**Table 1:** Clinical characteristics of responders and non-responders.

	Responders (n = 17)		Non-responders (n = 75)	
Gender (M/F)	10/7		58/17	
Age, years	27 (19–42)		28 (19–45)	
HBV genotype B/C	8/9		37/38	
	**Baseline**	**End of follow-up**	**Baseline**	**End of follow-up**
HBV-DNA, Log_10_IU/mL	8.1 ± 7.7	2.5 ± 2	8.5 ± 8.3	7.9 ± 7.2
HBsAg, IU/mL	14,091 ± 3,595	2,142 ± 823	22,175 ± 3,278	12,540 ± 1,705
Anti-HBsAg positive	0/17 (0%)	0/17 (0%)	0/75 (0%)	0/75 (0%)
HBeAg, COI	429.2 ± 113.8	0.24 ± 0.059*	6,162 ± 1,371	404.8 ± 60.6
Anti-HBeAg positive	0/17 (0%)	17/17 (100%)	0/75 (0%)	27/75 (36%)
Anti-HBcAg positive	17/17 (100%)	17/17 (100%)	75/75 (100%)	75/75 (100%)
ALT, U/L	217 (80–580)	21 (10–33)	218 (75–530)	124 (19–1107)
AST, U/L	108 (50–244)	30 (19–49)	117 (24–346)	39 (17–141)

^*^HBeAg negative was defined as HBeAg <1 COI. Unless otherwise indicated, values are median (range). Values for HBV-DNA, HBsAg, and HBeAg are median ± SEM. ALT, alanine aminotransferase; AST, aspartate aminotransferase; HBV, hepatitis B virus; HBsAg, hepatitis B surface antigen; HBeAg, hepatitis B envelope antigen; COI, cutoff index.
