# Supplementary material for: NKp30+ NK cells are associated with HBV control during pegylated-interferon-alpha-2b therapy of chronic hepatitis B
Source: Sci Rep. 2016 Dec 12;6:38778. doi: 10.1038/srep38778 (PMC5150634; doi:10.1038/srep38778)
Supplement: Supplementary Information [file srep38778-s1.pdf]

NKp30+ NK cells are associated with HBV control during pegylated-interferon-alpha-2b therapy of chronic hepatitis B

Xiaokun Shen, Binqing Fu, Yanyan Liu, Chuang Guo, Ying Ye, Rui Sun, Jiabin Li, Zhigang Tian, Haiming Wei

Inventory of Supplementary Information

Supplementary Figure 1. Schematic overview of selected patients.

Supplementary Figure 2. The gating strategy for NK cells and treatment with ADV had no impact on the total number of NK cells.

Supplementary Figure 3. The levels of NKp44, NKp46, Tim-3 and PD-1 expression on NK cells were not significantly different between the SR and NR patients during therapy.

Supplementary Figure 4. Comparison of markers of NCRs, cytotoxicity and activation during Peg-IFN-α monotherapy compared with Peg-IFN-α plus ADV combination therapy.

Supplementary Figure 5. CD215 (IL-15R) expression on different immune cell subsets in Responders and Non-Responders.

Supplementary Figure 6. Impact of differing therapies on the clinical parameters.

Supplementary Table 1. Response at the end of follow-up

Supplementary Table 2. Antibodies used for flow cytometry

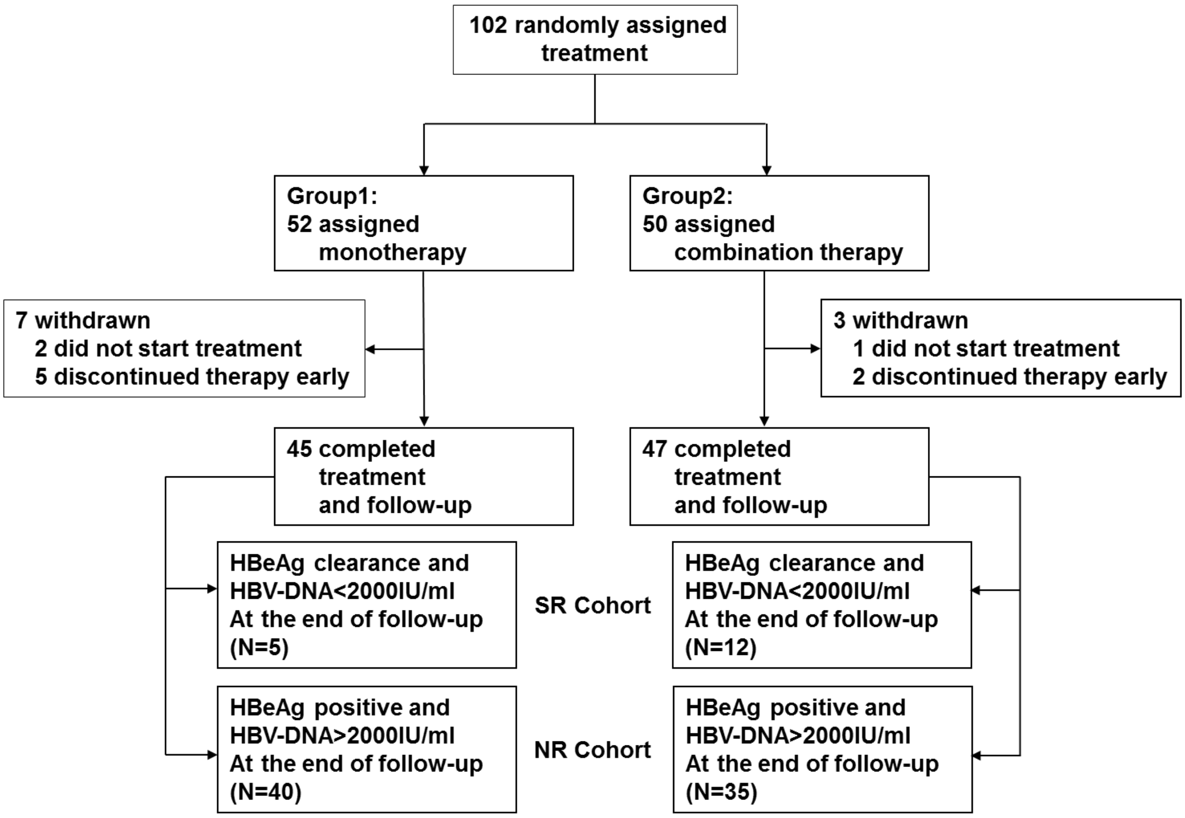

Supplementary Figure 1. Schematic overview of selected patients.

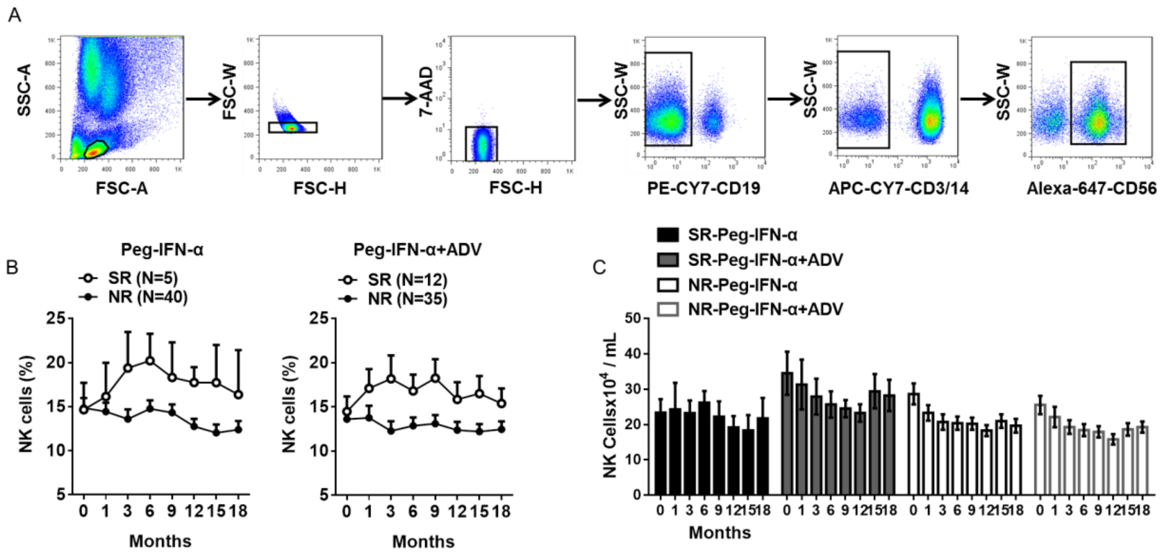

Supplementary Figure 2. The gating strategy for NK cells and treatment with ADV had no impact on the total number of NK cells. **(A)** Lymphocytes were identified by forward and side scatter, and single cells were identified. Next, dead cells (7-AAD), B cells (CD19), monocytes (CD14) and T cells (CD3) were excluded, and NK cells were defined as $CD56^{+}$. **(B)** The percentage of NK cells in the two treatment groups during the 12 months of therapy and 6 months of follow-up. **(C)** Absolute numbers of NK cells in the two treatment groups during the 12 months of therapy and 6 months of follow-up.

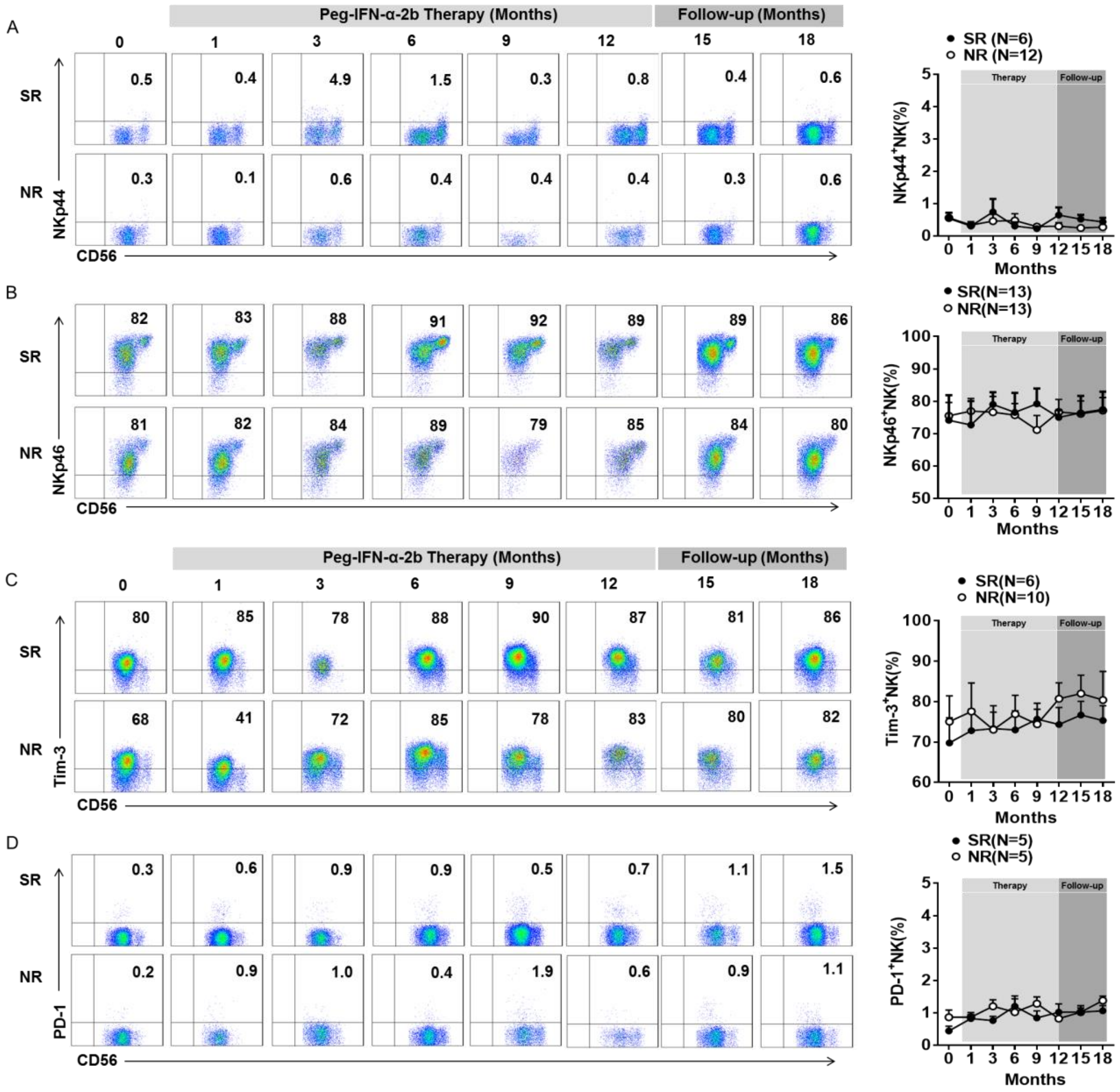

Supplementary Figure 3. The levels of NKp44, NKp46, Tim-3 and PD-1 expression on NK cells were not significantly different between the SR and NR patients during therapy. **(A, B)** A longitudinal analysis of the expression of NKp44 and NKp46 in paired samples from the SR and NR patients. **(C, D)** A longitudinal analysis of the expression of Tim-3 and PD-1 in paired samples from the SR and NR patients.

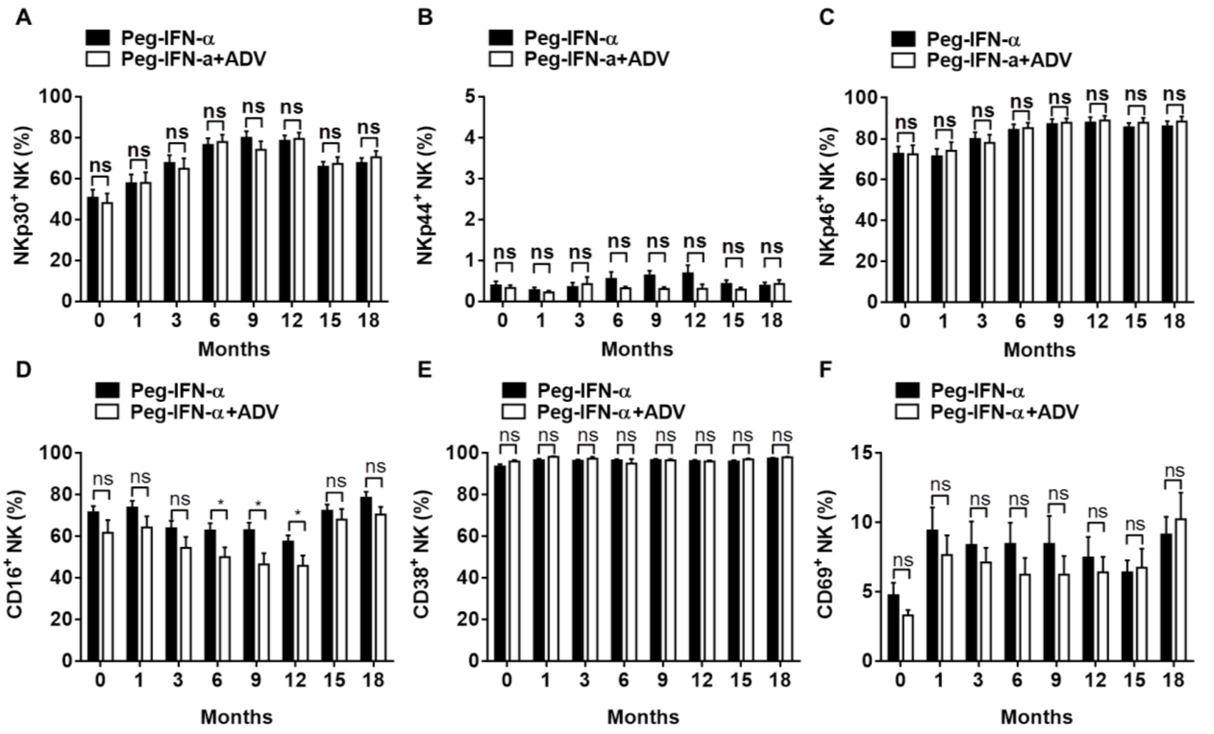

Supplementary Figure 4. Comparison of markers of NCRs, cytotoxicity and activation during Peg-IFN-α monotherapy compared with Peg-IFN-α plus ADV combination therapy. Percentage of: **(A)** NKp30$^+$, **(B)** NKp44$^+$, **(C)** NKp46$^+$, **(D)** CD16$^+$, **(E)** CD38$^+$, and **(F)** CD69$^+$ NK cells from patients in each treatment cohort. The results are expressed as the mean ± SEM. Significant changes are marked with asterisks, *p < 0.05.

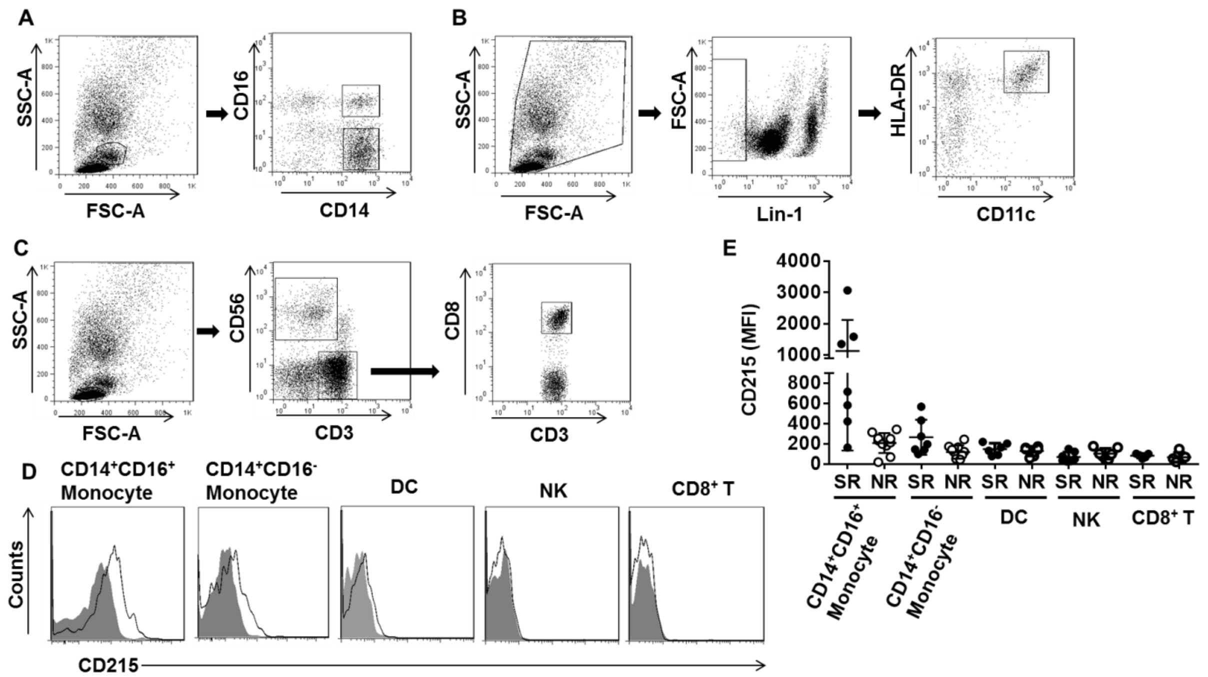

Supplementary Figure 5. CD215 (IL-15R) expression on different immune cell subsets in Responders and Non-Responders. **(A)** The gating strategy of pro-inflammatory $CD14^{+}$ $CD16^{+}$ monocytes and classical $CD14^{+}$ $CD16^{-}$ monocytes. **(B)** The gating strategy of myeloid dendritic cells. **(C)** The gating strategy of NK cells and $CD8^{+}$ T cells. **(D)** Representative FACS analysis of CD215 (IL-15Rα) surface expression on the different immune cell subsets, including monocytes, myeloid dendritic cells, NK cells, and $CD8^{+}$ T lymphocytes. **(E)** Mean fluorescence intensity (MFI) of IL-15Rα (CD215) expression on different immune cell subsets from the SR (black) and NR (white) patients.

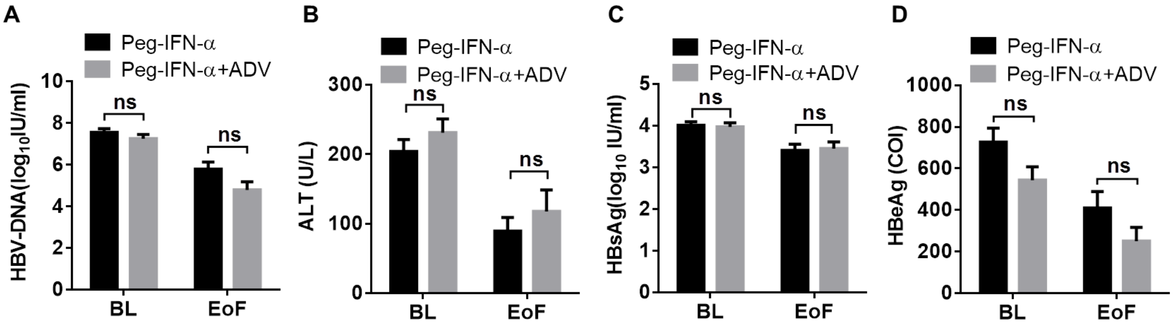

Supplementary Figure 6. Impact of differing therapies on the clinical parameters. Change in **(A)** HBV-DNA loads (log_{10} IU/ml), **(B)** ALT levels (U/L), **(C)** HBsAg titres (log_{10} IU/ml) and **(D)** HBeAg levels (COI) in the Peg-IFN-α monotherapy cohort compared with the Peg-IFN-α plus ADV combination therapy cohort. BL, baseline; EoF, end of follow-up.

Supplementary Table 1. Response at the end of follow-up

Treatment Response	Group of treatment	No. of Patients	% of Patients	*p* Value
HBeAg seroconversion[*]	PEG-IFN-α-2b	12/45	26.7%	0.442
	PEG-IFNα-2b+ADV	16/47	34%	
Sustained response[#]	PEG-IFN-α-2b	5/45	11.1%	0.075
	PEG-IFNα-2b+ADV	12/47	25.5%	

*** HBeAg loss and seroconversion to anti-HBe.**

[#] HBeAg seroconversion, ALT normalisation and serum HBV DNA < 2000 IU/ml.

Supplementary Table 2. Antibodies used for flow cytometry

**Specificity**	**Fluorochrome**	**Company**
CD3	APC-CY7	BD Biosciences
CD56	Alexa-647	BD Biosciences
CD56	Alexa-488	BD Biosciences
NKp30	PE	BD Biosciences
NKp30	Alexa-647	BD Biosciences
NKp46	PE	BD Biosciences
NKp44	PE	BD Biosciences
Ki-67	Alexa-647	BD Biosciences
IFN-γ	FITC	BD Biosciences
CD107a	PE	BD Biosciences
CD62L	FITC	BD Biosciences
PD-1	PE	BD Biosciences
NKG2A	PE	R&D Systems
TIM-3	PE	R&D Systems
CD14	APC-CY7	BD Biosciences
CD19	PE-CY7	BD Biosciences
7-AAD	PE-CY5	BD Biosciences
CD215	PE	Biolegend
Mouse IgG1 Iso Control	FITC	BD Biosciences
Mouse IgG1 Iso Control	PE	BD Biosciences
Mouse IgG2a Iso Control	PE	BD Biosciences
Mouse IgG1 Iso Control	Alexa-647	BD Biosciences
Mouse IgG1 Iso Control	PE-CY7	BD Biosciences
Mouse IgG1 Iso Control	APC-Cy7	BD Biosciences
Mouse IgG2b Iso Control	APC-Cy7	BD Biosciences
